# Korean autistic persons facing systemic stigmatization from middle education schools: daily survival on the edge as a puppet

**DOI:** 10.3389/fpsyt.2024.1260318

**Published:** 2024-03-28

**Authors:** Wn-ho Yoon, JaeKyung Seo, Cheolung Je

**Affiliations:** ^1^ Korean Research Center for Guardianship and Trusts, Hanyang University, Seongdong-gu, Seoul, Republic of Korea; ^2^ Social Welfare Institute, Sungkonghoe University, Guro-Gu, Seoul, Republic of Korea; ^3^ School of Law, Hanyang University, Seoul, Republic of Korea

**Keywords:** school bullying, stigma, autistic traits, objectification, authoritarianism, autism spectrum

## Abstract

**Introduction:**

Korean autistic persons who have endured an integrated secondary education system have been exposed to school bullying, causing trauma and stigma to them. It also blocks them from entering a tertiary education system and a decent work, resulting in a lower quality of life. However, research on how it affects autistic persons has not yet been conducted in Korea.

**Methods:**

Fourteen adult autistic persons in the Republic of Korea participated in the semi-structured focused group interviews. Their conversations were analyzed through qualitative coding.

**Results:**

The interview results show the rare voice of Korean autistic people. Although interviewees experienced physical, verbal, and sexual violence against them during the secondary education period, they could not get substantial assistance from schools and society. Interviewees agreed that bullying is inherent in the secondary education system of Korea, even in Korean culture. They experienced the cause of bullying being attributed to them as victims rather than perpetrators, and impunity is given to the bullying assailants. Early analyses of this article confirm that such experiences are combined with the sociocultural climate of elitism, meritocracy, and authoritarianism in the Republic of Korea.

**Conclusion:**

The study confirmed that the autistic person’s bullying experience does not come from the social inability of autistic people but the “profound” competition and discriminative atmosphere of the society. The result urges further studies on the bullying experience of East Asian autistic persons and the construction of Korean intervention strategies to prevent school violence against Koreans with disabilities, especially autistic pupils.

## 1 Introduction

Bullying, expressed as *Wangtta* in Korean and *Ijime* in Japanese, is an “intentional, repeated” (1) victimization of a person by others in thinkable various ways of humiliation (2) and objectification (3, 4), including abusive utterances, relational manipulation, exploitation (5, 6), physical hits, sexual harassment, and cyberbullying (7). Bullying can lead to eating disorders (8), obesity (9), further physical and psychological problems (10), social isolation (11, 12) or *hikikomori* (13), and even leads to victims’ murder or suicide (6, 14).

Most autistic people seemingly face school bullying from their “peers.” Park et al. (15) suggests that 67% of autistic secondary school pupils endure victimization, and 14% are related to perpetration and victimization in primary and secondary schools by meta-analysis. In addition, Maïano et al. (16) also suggest that 44% are related to victimization and 14% are related to perpetration and victimization. The result may be contradicted by Trundle et al. (17), who found the prevalence of bullying against autistic people at 44%. However, these results can be complementary because their analysis contained bullying in all ages. The results of meta-analysises suggest that most bullying experiences of autistic people may occur during secondary school.

Bullying against autistic people mostly occurs between the communication of autistic and allistic people. Autistic people seem to have a different interaction strategy due to the difference in brain neurology (18). Some autistic-suggested theories, like the double empathy problem (DEP) (19) and monotropism (20), support the theorem of neurodiversity. In addition, the neurodiversity model coincides with the human rights model of disability (21, 22), the legal approach to disability under international law, according to the UN Convention on the Rights of Persons with Disabilities (23).

The problem is that autistic perception and communication approaches and strategies are regarded as an impairment of social communication (24) or the “lack of theory of mind” (25). Therefore, what is going on with autistic students in secondary schools, which impacts significantly in their lives, has been ignored or has a low priority in autism research. For example, only 3% of research on autistic traits in America (2018) went to transition and adult issues (26).

Bullying in school against autistic persons and their negative experiences in the school affect their “prognosis.” For example, in 2022, only 16.8% of autistic pupils (402 persons) remained in regular classes in secondary schools in Korea; other Korean autistic students often go to special schools or special classes in schools (27). Therefore, most Korean autistic pupils cannot advance to the tertiary education system, especially graduate schools. Moreover, the employment rate of Korean autistic people in 2022 was 26.7% (28), much lower than the overall employment rate of 68.5% (29). Most autistic persons cannot advance into decent jobs or remain in low-salary jobs because they cannot get enough knowledge and skill(s) through the education system to work in companies, or many companies do not want to hire them (30). Therefore, most autistic people could not earn enough money to live well in the Korean society. Moreover, trauma and stigma resulting from bullying could trigger other mental health problems (10), a highly stigmatic disposition in Korea resulting in social exclusion (31).

Nevertheless, the research on bullying against autistic people is only at a starting point. For example, according to Park et al. (15:911), the number of research articles on bullying autistic people by January 2018 in English, Japanese, and Korean was 752, 227, and 20, respectively, which shows that academic concern lags in terms of the number of articles. Despite many researchers interested in autism in these countries, Korean articles focused on either autistic persons’ parents (32–34) or literature analysis (35) and quantitative analysis (36). The status proves the need for research on what is happening in the school life of autistic persons, especially on school violence against autistic pupils in their own words.

Therefore, we want to start answering this problem to find out what was happening in secondary schools in Korea. First, we want to clarify why bullying occurs from looking at the applied humanities and law studies viewpoint. Then, we will show the results from the focused group interview (FGI) with Korean autistic people.

## 2 Meaning of bullying against autistic people in the Korean society

### 2.1 Social environment where bullying takes place

Before discussing a Korean autistic person’s exposure to bullying, we need to understand the background that enables easy bullying against people with disabilities including autistic people.

Korea has a high-contextual (37) and “Confucian meritocracy” culture (38), which requires its people to be productive. The 12-year mandatory education, especially secondary education—constituted of middle schools and high schools—has been regarded mainly as preparatory courses for the College Scholastic Ability Test (CSAT) or *sunwng*, to go to “top universities” (39). The Korean government keeps CSAT day a special one, including making Notice to Airmen for blocking aircraft from airport runways and its airspace, during the hours when examinees hear Korean and English questions from speakers (39). To ensure that children and teens eventually enter higher-ranked universities, many parents require “their own” kids to learn a huge amount of knowledge before they officially enter even elementary school, especially in mathematics (39, 40).

The point system of CSAT utilizes the standard normal distribution (SD), which corresponds with the neuronormality (41) and medical model of disability. In the CSAT, examinees get higher scores when they are among the top 4% of examinees (the next grade is 11%) and get higher points if their standard deviation is higher. Under this system, examinees can get a third grade with just one failed question if more than 11% of the examinees in a test subject get a perfect score. Therefore, examiners must create extremely hard questions in the CSAT to make superiority and inferiority between examinees discernible, and the level of difficulty of CSAT has constantly increased to distinguish the “learning power” of examinees.

This “fierce competition” (42) is seen as important not only for the lifetime of an individual, but its importance reaches for family, community, and the nation. For example, many families see university as a predictor of their child’s position in the labor market, because admission to an “elite” university is assumed to ensure the highest career in Korea (42, 43). Moreover, the educational performance of schools has been thought to be evaluated with the average CSAT score of local high school pupils and how many pupils at a certain high school entered elite universities (44). The Korean government also refers to international educational exam results, such as the Programme for International Student Assessment as indicators of a country’s future (39).

Therefore, during the high school period, most pupils are forced to live an austere life to enter a better university. One of those practices is the night self-study or *yaja*, which is *de facto* mandatory studying (45, 46) that makes pupils study until 9 or 10 p.m. (47). During *yaja*, teachers round classes to supervise them to see whether they are studying or not. They punish any pupil who is away from their studies (45). Recently, some local education boards lifted night self-study as a mandatory program, but some schools and parents continue this program, fearing lower scores for their children (46).

Moreover, to make their kids more competitive than others and achieve a successful lifetime, most parents send their children to shadow education or *hagwons* (48). Many children participate in advanced education in a *hagwon* to preempt a higher place in the unending competition (40, 43, 48). According to a recent survey, 78.3% of pupils go to *hagwons*, and their parents spend around 4,100 USD equivalent value in a year (49), which has unexpectedly increased in some years (43). Moreover, overall expenditures for shadow education in 2022 are 19.9 billion US dollars (26 trillion won), which is 1.1% of the GDP in 2022 (49). In other cases, some schools operate small dormitories for honor class to encourage pupils in higher test points to “immerse in study” (40). Some schools oblige all pupils to enter the school dormitory and impose inhumane policies without consent or free will, which violates human rights (50).

These institutions affect Korean pupils negatively. According to Joo et al. (51), high school pupils in Korea usually go to sleep after midnight and wake up before 8 a.m., sleeping only 6 h a day. However, many pupils are encouraged to sleep less because they assume that to “save time for sleep” until some days from CSAT day is indispensable for those who want to advance to elite schools like *seoyeongo* (52), which is limited to 10,000 spots (53). The unofficial four-character idiom *sadangorag* (“four-pass five-fail”) defines their lifestyle (40): An examinee must sleep only for 4 h on weekdays to get into elite universities.

The negative effect of competitive education is clear: stress from learning (47) and excessive daytime sleepiness (51). Long learning time also lowers life satisfaction and school adjustment and induces depression (54), runaway, and suicidality (47). Most of all, the free will of pupils is ignored in this kind of mandatory education system, which violates the human rights of children (44).

In this very competitive system, anything disturbing their study, which is the reward strategy for the hardship of study or solution for the stress-resolving solution, is strictly prohibited. Especially, anything that would make secondary school pupils escape from “linear repetitions” (55), which, as Henri Lefebvre described as a *détournement* (56:17), becomes a sin.

For example, in 2011, the Korean government under President Lee, Myeong-bak enforced the reformed Youth Protection Act, supported by parent organizations, which imposed internet game providers blocking access from teenagers from midnight to 6 a.m. (57); the policy officially repealed in 2021 with unsuccessful effects, but it shows the authoritarian view of pupils, which ignores their human rights (58).

In addition, families, schools, and society condemn “examinees” enjoying cultural contents, such as K-POP (59), webtoons, web novels (*websoseol*), animé and *manhwa* (comics), and cosplay (60), because they oppose instrumentality of life (56). Indeed, some hook songs are called as a prohibited song for *sunwng*. Certainly, many pupils deviate from the prescribed meaning (56:21) and objectivity, but then their families and authorities punish them, forcing them to “stay still” until they graduate high school.

Moreover, some researchers even connect (social) communication with friends or using smartphones to “depression and suicidality” (61) because they reduce the time which is not related to “productive behavior”: Learning, eating, and sleeping are only allowed in high school period. This is the modernistic, and authoritarian viewpoint of life, and it has value for criticism in social studies.

The harsh education environment puts teenagers under utmost stress, which induces anger and rage (62, 63). In addition, many pupils construct a negative view of the society and the education system, which increases their rage (64).

Many Korean pupils in secondary education schools have extensive learning time, and, to them, a confrontational strategy by pupils is considered a sin; they are eager to search for objects to relieve stress, and it is bullying because it is the easiest way to relieve stress. This is the point that is usually overlooked in the (autistic) life experience of people in Korea.

### 2.2 Relationship between bullying and stigma in autistic people

As mentioned in the introduction, there is a strong link between autism and bullying, and the severity of the emotional impact has been documented in numerous studies (65). Hence, why is bullying so prevalent against autistic people? To find out the connection between bullying and autistic people, we want to take back from the notion of stigma, which has been confirmed by Erving Goffman (66), and the theme of this special issue.

Goffman’s concept of stigma is often understood to be limited to the phenomenon of discrimination itself, which occurs because a person with a disability has certain “attributes” that are understood by society as “stigma” and because the members of the society notice those traits (66). However, if we re-read Goffman’s discussion, we can see that the stigma phenomenon is a social construction that is shaped first and foremost by a society’s cultural system and its power system.

According to Goffman, stigma is only constructed through communication with others. For example, a person with disability who does not go to school may be protected by his/her family (66:33). Police officers, on the other hand, can identify suspicious people by sight and use techniques such as framing and blackmail to incriminate them (66:75–76). Social stigmas, especially those imposed on people without disabilities, can be removed when the members of the society who knew them do not exist when they succeed in fleeing from their communities. Therefore, Stigmas can be reinterpreted as being formed when peers or communities find a reason to harm a person and then harass them for it, and the group accepts and shares this collective memory.

An important point is that stigma closely influences and is influenced by collective empathy (67) and operates particularly strongly where it is imbued with prescribed social meanings (68). For example, one might compare who is more actively engaged in society today, between contemporary people with disabilities and those of the “enlightenment era,” where, as Foucault pointed out, the body was domesticated as a docile body, controlled by the power (69), and the society and intellectuals were controlled (70) to drive social progress. It should also not be denied that this exclusion was deeply connected to social evolutionary theory and eugenics, which sought to separate the “abnormal” from the “normal” to develop a “healthy” society (71).

From the late 19th century to the mid-20th century, American society, at the height of its modernity, had a policy of only accepting immigrants whose bodies and behavior did not exhibit “signs” that could be interpreted as stigma (72) to keep “unhealthy” outsiders out, and these noticing people were everywhere, spying on audiences at the entrances of theaters and shops and arbitrarily removing them (66:70). At the same time, the United States had a policy of thoroughly objectifying Black people (73): The effects of this racism continue to stress Black people in American society today (74).

Understanding this historical context, therefore, reveals that stigma is not expressed by the individual with stigma but by others who are “enabled” to read the individual’s difference as an impairment and interpret it as a disability. This fact supports the social model of disability and, at the same time, challenges the medical model of disability, which is the dominant ideology through which autism is perceived.

Autistic people are officially represented as “persons with autism spectrum disorder” (24), which suggests that they are in disordered (therefore, that is out of the “order”) status (75). The diagnostic criteria for this abnormality include intense interest and an inability to respond to “Social-emotional” communication and combines visual information other than speech (24). However, the diagnostic criterion is not fair, because the criteria are clinical views and prescribed from non-autistic perspectives such as the theory of mind or eye contact (25, 76), far from the social model of disability.

For example, the DEP by Milton (19) suggests that the social initiative problems of autistic people are not created because the autistic trait is not a deficiency of social communication abilities, but allistic (or neurotypical) people are unable to communicate in the autistic or neurodivergent communication system (77, 78). There is evidence supporting the DEP that shows that groups with and without autism have the same degree of social communication in each group (79–82).

However, because the way that autistic people communicate is different from what society has constructed, neurotypicals soon discover autistic people despite their masking strategy (83, 84), and these differences become a major reason for people without disabilities to withdraw from relationships with autistic people. Unfortunately, Goffman finds that discrimination based on difference is not only perpetuated by people with disabilities but also by various other stigmas of the time. According to Goffman, some people with disabilities could only exist in “public” as minstrels or clowns, and they were “encouraged to have a distaste for those of his fellows … without actually making a secret of their stigma … to show that despite appearances they are very sane, gentlemen deviants, nice persons like ourselves despite the reputation of their kind” (66:110–111). Those who ignore this “air” (85) and enter the space of the “normal” are subjected to the gaze of exclusion, and the stigmatized person is required to exist in a space where the “normal” is unable to see them (66:119-121).

Of all the ways that peers reject this “notification,” violence is the most efficient, leaving an indelible mark on the victim while benefiting the perpetrator. Autistic people are less likely to have adequate coping skills for bullying (86), so pranks may be escalated to the point where they become gaslighted. Therefore, bullying and peer rejection of communication are risks that should be actively controlled, as they not only prevent well-meaning social communication initiatives in adolescent autistic people but also increase stress and the likelihood of psychiatric complications (65, 87).

However, we find indifference and lack of action on the part of educational authorities toward bullying against autistic people and the absence of sufficient methodologies for dealing with bullying. Here, we can find a system of stigmatization through bullying that prevents autistic people from participating in society. Autistic people who enter the secondary education system become targets of bullying because of their autistic behavior to regulate their stress or intense interest—neurodivergent expression. As the bullying continues, autistic people are disrupted from learning necessary for tertiary education and may end up being segregated in special classes or special schools, or they are forced to endure the violence on an ongoing basis. As a result, school violence against autistic people can be interpreted as being used as a tool to visualize or justify the stigmatization of autistic people.

### 2.3 Bullying in the law system of the Republic of Korea

School bullying attracted public attention around the turn of the 21st century in Korea. Then, the Act on the Prevention of, and Countermeasures Against, Violence in Schools (hereinafter the School Violence Prevention Act) came into force on 30 July 2004 (88). This act provides that perpetrators shall be subject to various sanctions or countermeasures, ranging from ordering an apology letter to victims to expulsion from schools by the decision of the autonomous committee for countermeasures against school violence (hereinafter the *hakpogwi*) established in individual schools, which is composed of teachers, representatives of parents and other experts. Such sanctions are supposed to deter school bullying because they may be recorded in the school performance record of the perpetrator. When school bullying conforms to either crime or juvenile delinquency, perpetrators may be subject to sanctions by the Juvenile Act. All these measures are punishment, which schoolteachers and police are usually reluctant to resort to as punishment for school bullying because it is likely to damage university entrance competition, which parents and children desperately cling to (89). Because of the reluctance of the committee to take appropriate countermeasures against school violence, the reformed School Violence Prevention Act in 2019 transferred the function of the committee to the level of the district office of education. Thereby, the sanction function is supposed to be removed from individual schools. But, still, the principal of the school has the right to close “slight school violence” cases (90, 91).

On the other hand, social welfare measures to prevent school bullying are lacking. Because perpetrators are likely to be victims of child abuse and neglect as well, a child protective agency might intervene to provide appropriate support and counseling to parents and children to prevent and mitigate potential school bullying as the consequent effect of abuse and neglect. However, such interventions are yet to be implemented.

## 3 Method

### 3.1 Procedure

To understand the bullying experience of autistic people in Korea, we adopted the focused group interview with ethnographic methodology. All autistic persons who identifies them as autistic, and speaks Korean, regardless of diagnosis, were eligible for participation in this research. Most participants were contacted through advertisements that were distributed in autistic and neurodivergent advocacy groups. Furthermore, we tried to find more potential participants in other areas, through the advertisement to welfare centers, organizations for people with disabilities, and academic associations in Korea.

The research process was designed to be two-step: Participants can select to come to Seoul or connect via an online meeting service, according to their allowance. After getting the informed consent in (electronic) documents, all participants will receive the semi-structured focused group interview, in which two to three autistic persons answer pre-defined and instant questions for 1 h and a half to 2 h. Examples of fixed questions are in **Table 1**. Then, selected participants who would have resilience or further important testimony move on to another focused interview.

**Table 1:** Excerption of fixed interview questions.

• Exposure to violence and schooling experiences in secondary education - How did you feel when you first went to school? - Do you remember the first time you were exposed to school violence? - Do you remember any words from the other pupils? - What other challenges did you face in school? • Coping with bullying - How did you deal with stress at that time? - Have you ever told a teacher or family member about bullying, and what was their reaction? - Did you have opportunities to consult about your bullying and school life? - Have you ever used a program such as “Letters to and from” or “Wee class” and what was your experience? • Desire for support for bullying - What did you want most when you were a victim of bullying? - Based on experience, what interventions are needed for autistic students who are still experiencing bullying? • What does secondary education mean to you? - What is the significance of secondary education in your life? - Where would you go if you had to go back and choose between a special school and a mainstream school? - What do you think where is the best school for autistic people?

The research could induce some traumatic reactions by the flashback of their bullying memories. Therefore, to verify the ethicality of the research process, the research process got ethical approval from the Institutional Review Board of Hanyang University (Approval No. HYUIRB-202303-005).

Fourteen participants participated in the first round, through three offline interviews and three online interviews. However, after we ended the first round, we found the utterances of participants constituted a theoretical saturation, because the number of participants was over the suggested sample size and the interview results constituted new concepts (92). Therefore, we received revision approval from the Institutional Review Board for closing out the research.

The interview audio files, which were recorded with the consent of participants, were transcribed into Korean, and, then, two reviewers reviewed the transcriptions independently, according to the triangulation methodology (93). Reviewers constantly find the meaning units from the transcriptions and sort them into categories according to their relativeness. The categories and the analysis of each researcher were compared in the meeting of researchers and triangulated with other reviews. After the reviewing, one reviewer compromised it as a document.

After the Korean analysis results were out, the results were circulated to participants to confirm the accurate reflection of their own experiences. All participants agreed to the results, with some edit requests. The request was applied to the result of this article.

### 3.2 Participants

Fourteen autistic persons gave researchers documented consent to participate in the research. The demographic characteristics of the participants are shown in **Table 2**. 

**Table 2:** Demographic characteristics.

Categories	M or N (SD or %)
Age (n = 14)	30.29 (6.27)
Gender (women)	4 (28.6%)
Race/ethnicity	
Korean	14 (100%)
Education status	
Dropout of high school	1 (7.1%)
High school graduated	3 (21.3%)
University student	2 (14.2%)
Bachelor**’**s degree	7 (50.0%)
Graduated school student	1 (7.1%)
Disability registration to the Korean government	
Autistic persons with ASD registration	4 (28.6%)
Autistic persons with mental disability registration	2 (14.3%)
Autistic persons without recognition	8 (57.1%)

All participants reported their autistic identity. However, only four persons were regarded as “people with autism spectrum disorder” by the Korean government, which is due to the rigid medical criteria of the Korean national disability registration system (94). Rather, more participants were self-diagnosed persons because they are unable to get a diagnosis for several reasons.

To secure the anonymity of the participants, we randomized each person’s number in the following results.

## 4 Results

The results have been summarized into five general themes. These are (1) mainstream society that draws the line at outsiders; (2) surviving alone on the edge of school violence; (3) autistic traits as a playing object in malicious play; (4) bullying grows out of ableism and an authoritarian environment; and (5) the suggestions from personal experience.

The participants described their bullying experiences as struggling to survive on the fringes of the school system and enduring physical and verbal abuse such as bullying, ignoring, ostracizing, ridiculing, and teasing from mainstream social groups who drew the line to them as outsiders.

For them, school is a horrific hell, a traumatic space that gives them nightmares: They became toys for malicious play just because they “have autism.” Participants found that the perpetuation of bullying in schools is fed by the nourishment of ableism, meritocracy, and authoritarianism, which suggests that bullying experience intertwines with meritocracy and authoritarianism in mainstream society. Concretely, in secondary schools, mediocracy is supported by schools, which routinized corporal punishment and gives impunity to perpetrators who are stronger than victims. Furthermore, schools had an authoritarian social culture that found problems in the victim rather than the perpetrator. The Summarized results are in **Table 3**.

**Table 3:** The result from the FGI.

Subject	Sub-category	Meaning unit
**Mainstream society** **draws the line at outsiders**	**People who stigmatize**	Being treated like a psychotic Being teased as a *Byeongtta* (“disable-bullied”) Become a target of rumored bullying by middle school Teased and ostracized for being disabled
	**Logic of inferiority**	Assaulted for being an inferior student who could not go to university Beaten because they were dwarfed by their physique Beatings for being disabled and not having a father
	**Bullying and harassment based on disability prejudice**	Called *aeja* (“disabled”) because of low intelligence Ignored by teachers for having a disability, which led to students ignoring them as well
**Surviving alone on the edge of school violence**	**Surviving 1 day at a time**	Getting through the day without the strength to fight back Imagining a bleak future for the bully Imagining a hero to save me
	**Schools in trouble**	Teachers looking the allistic pupils A classroom teacher’s lack of intervention leads to hostile relationships with classmates Victims of bullying are seen as pitiful person with disabilities School administrative support system that is only display for showing Lenient school punishments for perpetrators of bullying
**Autistic traits as a playing object in malicious play**	**Instrumentalization by bullies**	Being beated became daily routine I was so ashamed of the way bullies used to harass and placate me A classmate who bullied me in my own home with pride
	**Instrumentalization by perpetrators**	Being treated as a puppet Being used as a punching bag for my classmates’ emotional outbursts Being hit repeatedly for looking up
	**The logic of difference is used as a justification for bullying**	The stimming becomes an excuse for bullying My unusual hobbies (interests) are used as an excuse for bullying Classmates who exploited my fastidiousness to emotionally abuse me Speaking differently than my peers is used as an excuse for teasing
**Bullying grows from ableism and an authoritarian environment**	**Bullying as a parasite on meritocratic nourishment**	Schools coddle academic perpetrators Oppressed students in a meritocratic school system use students with disabilities as an outlet for stress Targeted by perpetrators because of their inability to study
	**Bullying as a parasite on authoritarian societies**	A school culture where corporal punishment is the norm serves as a justification for bullying Older generations dismiss bullying as a prank Mainstream social groups assume that children are victims of bullying because they are unable to study and are weak
**Suggestions from the experience**	**Strengthening** **the legal system**	A weak legal system sends the message that “You can deal them with anything” Strong penalties for perpetrators of school violence are important Confidentiality for complainants and suicide prevention systems for victims are needed
	**Increase disability sensitivity** **in mainstream communities**	Training on disability sensitivity is needed Strict separation of perpetrators of bullying is important Teachers need to be trained in disability sensitivity Mainstream society needs to be educated on how to deal with people with ASD
	**The necessity of the** **person-centered** **self-advocacy system**	Service and support system for Neurodivergent persons without registration Training programs that give conversations with allistic persons Advocate to stand up for me The emotional community where one connects with other people The diversity-respected school system where I can feel empowered
	**Access to a supportive, professionally led counseling system**	Reformation of 'Wee project' that supports us from our perspective Need for a psychological counseling support system centered on specialists (psychiatrists) Need for a professional support system to prevent the development of interpersonal avoidance, schizophrenia, and depression due to mental trauma caused by school violence

### 4.1 Mainstream society draws the line at outsiders

Participants in the research remembered that the first thing that came to mind in their bullying experiences was people who labeled them as disabled. They recalled that they started to recognize their disability when they were called “mentally weak, sickly, and disabled”, were ridiculed, and were ostracized by those who labeled them. As a result of this ridicule, teasing, and harassment, the research participants cognized that they were outsiders who could not be included in mainstream social groups. They remembered the experience that started in elementary school, and, as they moved to middle and high school, the level and methods of bullying became more intelligent and sophisticated.

For the study participants, bullying was explored as a never-ending nightmare that continued uninterrupted from elementary school to middle school to high school. Most of the participants in the study experienced verbal abuse from the mainstream social group as early as elementary school:

“I got the most bullying in middle school. I cannot stop the list of them, let’s just take one. When I went somewhere, they teased me by saying *byeongtta*. The meaning is stupid (*byeongsin*)—outcasted (*wangtta*). They teased me using those words.” (P7)

The verbal abuse signals the notification of difference: “You are not like us.” The attack fulfilled the role that ostracizes the outsider who cannot be included in the mainstream social group. Through the signal and ostracism, the study participants felt that they became passive recipients of warnings from the mainstream social group rather than they could find their identity. They also found that they were “trapped in a caged structure” where their disability was constantly recognized and confirmed by others and outsiders.

In addition, participants reported that perpetrators stressed their inferiority to perpetrate violence against them.

“During the higher grades in elementary school, my father passed away. [peers] said, ‘Did you have a father?’. Every year, I experienced school bullying, from the first day to the last day of school.” (P9)

Their utterances further include not being able to go to university (P8) and having a smaller body size, showing the underlying logic of inferiority. It is also closely linked to what mainstream social groups consider to be the social markers of success, such as doing well in school, being physically superior, or coming from a rich or high–social status family.

Finally, they experienced ableism constantly throughout their schooling. Even when they talked about their hopes and dreams, like going to the Military Academy, mainstream social groups made fun of them or dismissed them as the future jobless, because of their disability. The participants described themselves as the oppressed person who had to live with disability hatred in the culture of mainstream social groups.

### 4.2 Surviving alone on the edge of school violence

Participants described that they survived alone on the fringes of school violence. They did not have any power to stop bullying against them. Moreover, participants could not have comfortable experiences, because some parents bothered them even in the family (P6), forcing them to survive: Their coping way was imagining a bleak future for the bullies (P3) or imagining a hero to save them.

“Passing middle school period, what I desired was a hero. One of my desires was it. I wanted to become a member of the hero narrative, to rescue myself in this status: A prince. The prince. That prince, I always have had an imagination that superhero save me.” (P13)

However, the hardest thing was the school: They thought the school itself was problematic. Schools were busy trying to please the mainstream social groups by giving lenient punishments to the bullying perpetrators while viewing victims as pitiful people with disabilities. They felt no advocacy system in the school could speak for them.

“In an elementary school, there was a system of ‘praise point passbooks.’ [One day the teacher] openly gave them +20 points just because they have been continuing their friendship with me.” (P2)

In addition, the clumsy intervention by teachers against the bullying made hostile relationships with peers and autistic pupils (P2 and P6). They felt that the school administration dealing with bullying was just for show, and there were no schools that advocated for victims, only problematic schools existed.

“I was pushed to the TV cabinet and just kept being punched in the head and the forearms, and I was almost beaten to death with one punch, and that was before the summer holidays of my first year. As the punching continued, my homeroom teacher and their homeroom teacher came, and their teacher punished them. Even though their teacher was expected not to give them a grade, the teacher just made them clean the classroom for a week, and the next year they were in the same class as me.” (P3)

The main reason for the forced survival of study participants was there was no advocate for them during their lives: neither in school nor in the family. In other words, they were forced to survive on the edges of school violence due to the absence of an advocacy system.

### 4.3 Autistic traits as a playing object in malicious play

Participants found that their traits were objectified and instrumentalized for bullying perpetrators’ malicious play, along with full teasing and ridicule with stigmatizing labels such as “mentally ill,” “sick,” and “disabled,” which came from the mainstream community. They remember that they lived with the bullying at schools every day: Some of the perpetrators even invaded their own houses.

“[The perpetrators] came to my house, what were they doing in my house?…If I upload that to SNS like Twitter, if they do like this, they will be ostracized almost. It’s a hamster, before me, they throw my hamster heavily, to kill [my hamster], not in their house, but in my house.” (P1)

Some of the most difficult memories of bullying for participants were times when they were instrumentalized by perpetrators. Perpetrators utilize stimming behaviors, literal understanding of meaning, intense interests, obsessive-compulsive behaviors, and different sensories, which are part of the autistic traits (95, 96), as a trigger for bullying.

“When I and my peers go somewhere, or I buy *tteokbokki* for them, then we get along anywhere. Let’s take an example. If the friend said to me: ‘How’s using this big bear doll instead of the umbrella?’ Then, I waded outside hugging the bear during huge rain.” (P5)

“There is a memory in elementary school, I just sat on my chair and kept writing sketchbook-like notes with strange figures and numbers. Then, they got the note and teased me [viewing the note].” (P2)

“Usually, kids have a way of understanding things like reading atmosphere or context. And they could notice differences between jokes and truth. But I couldn’t do that, that point made me the object of bullying when I was in elementary school.” (P12)

Perpetrators treated participants as punching bags, playthings, and emotional outlets for their play or playfulness. They described being treated as puppets or seen as someone who deserved to be hit or sexually harassed (P12) because they were different. Moreover, these experiences were associated with social withdrawal, difficulty in interacting with people and maintaining trusting relationships.

“[They] were making fun of me by drawing me as the subject of obscene graffiti, and when I made bad feelings, they got violent and punched me in the face. Someone who did not know how to control his/her sexual impulses embarrassed a classmate, and I got punched in the face, and from that, that’s when most of the boys started using me as a punching bag.” (P13)

This bullying pattern was important because it highlights how autistic people are perceived. When bullying perpetrators belonging to mainstream social groups treat autistic people as emotionless, objects of amusement, or instrumentalized them, it is more than an individual aberration. The undermining of the dignity of autistic individuals and their objectification as instruments of malicious play is a point where the fundamental problem of school education in Korean society is rooted, meaning that a more macro-level approach in a social structural context should be required.

Perpetrators of bullying from mainstream social groups were armed with a justification for the violence that they were different from the rest of the community. The difference is an instrument of violence against the research participants.

### 4.4 Bullying grows from ableism and an authoritarian environment

The social context of the participants’ experiences of bullying was two axes: (a) meritocracy and (b) authoritarianism.

First, study participants described that, during the coping process, they experienced unfairness, including being treated as idiots by their classmates or teachers protecting the perpetrators. They cited the entrance examination–orientated education system as the reason for the school culture that produces school violence. Influenced by the Korean social climate that favors and respects meritocracy, teachers regard students who score well as model students, and they may not have deviant actions or cause trouble but disregard those who are not good at studying. Therefore, the natural perpetrators of bullying are those who study well and are more respected than the victims of bullying who do not study well, which blocks the victims from receiving adequate support from bullying.

“The teachers took the perpetrators’ side, because the victim did not study well, and the perpetrator was a model pupil.” (P6)

Participants experienced bullying, were ridiculed, and were physically abused in a cultural climate that stigmatizes those who do not get good points as “stupid” (P10) due to the mainstream social mechanisms that have formed social standards and indicators in the center of those who succeed in elite universities. Moreover, one participant was scolded because they wept, which used to be prohibited in the Confucius society.

“I cried a lot, a lot more than other students of my age: there were so many things to cry about. And then other teachers or my parents would take it very badly, and they would give me orders, instructions, to be more mature because I wasn’t mature enough.” (P13)

However, participants mentioned that their bullying experience was due to the CSAT-oriented Korean education system, where all pupils experienced CSAT stress and therefore used themselves as an outlet for entrance examination stress. This suggests that school violence is parasitic and reproduced by the nourishment of meritocracy among mainstream social groups. Participants perceived that, because the Korean society climate supports the logic of going to an elite university to become a successful person, the perpetrators, who are unable to resist this logic and are suppressed and controlled at home and school, instrumentalize bullying by targeting their autistic classmates as the victim to relieve their stress.

Second, participants pointed out the authoritarian social culture of Korea that justified corporal punishment (97) and inhumane communication at secondary schools, which international law prohibits (98). Participants reported that the school culture and atmosphere, which justifies corporal punishment for students, gives perpetrators of school violence a justification for their bullying.

“It was a time when there was corporal punishment and all that kind of stuff, so it wasn’t strange for violence in schools and its spaces to take place. So, it’s kind of a cheesy thing to say. What are kids going to learn from that?” (P2)

The research participant referred to these behaviors as a microcosm of the society. This attitude was also reflected at home, where family members blamed the research participant, who was a victim of bullying, for the cause of bullying.

“They didn’t believe it, because they said that students who are good at studying never bully others. And when I talked about my bullying, everybody blamed me: they weren’t blaming the perpetrators.” (P13)

This phenomenon of placing the blame on the victim rather than the perpetrator was explored as a parasitic effect of the Korean society’s authoritarian culture, in which the strong are empowered and recognized (P6). Participants thought that the prevalence of corporal punishment and control over the people of the nation during the Korean authoritarian governments became the soil of reproduction of bullying in the Korean society. In addition, they referred to the social viewpoint that dismisses bullying as fun, and mainstream social groups that assume that those who could not study well or were weak were looked down upon and trampled upon. Whoever’s exam results are not good or weak, they are likely to be bullied.

“Adults said that they were just having fun with me because they wanted to play. They’re having fun, so why am I not happy? So, if I don’t like it, who am I to judge them? It was my thought.” (P4)

### 4.5 Suggestions from the experience

During the interview, participants suggested some coping methodologies: (a) strengthen the legal system, (b) increase disability sensitivity among mainstream social groups, (c) establish an autistic-centered self-advocacy system, and (d) establish a counseling system by professionals.

First, participants wanted to change the legal system regarding school bullying. Participants stressed that a current weak punishment system for bullying sends the message to perpetrators: “You can deal them with anything” (P13). They feared that the allowance of school gives legitimacy and perpetuates bullying. Therefore, they want strong punishments for perpetrators of bullying and to separate victims from the perpetrators, when the event is detected.

“Even when the *hakpogwi* was called, the [punishment] in the school remained as education volunteering, one week of classroom cleaning, writing apology letters, and making apologies in the document. If the countermeasures against school violence had been fortified the same as now, teachers would have received a disciplinary measure for the concealment [of bullying], and [perpetrators] would have gotten additional punishment.” (P3)

Moreover, they suspect that the Korean social climate does not favor confidentiality for those who report bullying. There is a report that teachers made public the bullying report to the class through homeroom time (P7). Therefore, they requested confidentiality for those who report bullying and a system to prevent victims from committing suicide. The system would be accomplished by thoroughly separating perpetrators and victims of bullying, but they did not experience that kind of help during their secondary education period (P13).

Second, participants mentioned how schoolteachers intervened by asking the mainstream community to pity students with disabilities (P7): They suggested enhancing disability acceptance among the mainstream community to decrease school violence. This is a point that is closely related to the mainstream community’s inadequacy of education for autistic people.

In addition, the disability sensitivity of mainstream social groups should be raised, and schools should play a role as a support system for service programs such as social skills training and dialogue training for situations where autistic people have difficulty communicating with people without disabilities. Participants in the study wanted a diverse school where they could feel empowered and had an advocate on their side.

Third, research participants identified the need for a person-centered self-advocacy system as a response and solution to bullying. Participants mentioned the need for a support system for undocumented people with neurodivergence (P3), a self-advocacy support system to empower them (P14), access to specialized services to support their difficulties in communicating with allistic people (P1 and P3), an emotional community to connect with people, and a diverse school system to empower them.

Finally, participants in the study mentioned a specialist-based counseling support system on school violence. They mentioned the Wee class, an education program for separating bullied students in other classes could not help because it caused side effects such as stigmatization (P6). Therefore, they need support from the perspective of the victims: They reckoned that psychiatrists with expertise in neurodiversity would help, rather than psychological counselors.

In addition, they reported the mental trauma caused by school bullying is extremely painful due to avoidance of interpersonal relationships, schizophrenia, and depression (P4, P8, P9, and P14). It was explored that the establishment of a counseling system centered on experts is also necessary in terms of preventing these problems.

“I don’t even like to be around people because I don’t have any good memories of synergy with people, so I can’t fit into society and now I’m almost like a *hikikomori*, and now I’m studying for my GED. I meet people, but if I meet them publicly, I don’t meet them privately, so honestly, I feel comfortable with that.” (P9)

## 5 Discussion

The results are consistent with the findings of the literature review on bullying against autistic persons to which this study was pre-referenced, thus reaffirming the objectivity of the result.

First, the root cause of the bullying was autistic traits. Autistic behavior was perceived as odd because it deviated from the highly structured rules required in the high-contextual Korean society. Korean society, along with other East Asian countries (99), values the collective identity (37, 67) and has a high tendency to downplay behaviors that are visibly different from mainstream groups, influenced by allocentrism and self-other similarity (100) and cultural stereotyping (101). Therefore, autistic people have lost their Confucian *raison d’être* and are easily objectified, which further contributes to the stigmatization of autistic people in the Korean society.

It may have a relationship with autistic people’s difference in cranial nerves of the brain neurodiversity (18). Some autistic-suggested theories, like the DEP (19) and Monotropism (20), support the theorem of neurodiversity. The problem is that neurodivergent perception and communication approaches and strategies are regarded as an impairment of social communication (24) or the “lack of theory of mind” (25) because they are no need for eye contact between autistic persons (81, 102). Therefore, it is usually considered a rude attitude to Korean allistic persons, which could justify “the correction” to unite autistic people to allistic persons: to keep neuronormality (41).

Second, the findings suggest that school bullying against autistic people is a social product rather than an individual fault. While participants reported trauma from the bullying, they also indicated that the roots of this violence lie in a variety of background factors, including societal discrimination against autistic traits, competitive schooling, and violence by schools (98). However, the Korean School Violence Prevention Act focuses on redressing school violence instead of preventing it and punishing perpetrators instead of providing relief to victims of school violence. In addition, the Ministry of Education has excluded pupils with disabilities who attend mainstream classes from the policy targets of the Comprehensive Plan for the Protection of Human Rights of Students with Disabilities released in 2018 (103). Therefore, the Korean government and society are also responsible for the prevalence of school violence against autistic people.

Third, there is a need to develop a distinct approach to autistic people outside of the Korean welfare system. In Korea, social services for developmental disabilities are provided for focusing on persons with intellectual disabilities, and there is still a lack of awareness about the bullying experiences and domestic abuse experienced by autistic people. However, it is known that the stigmatizing and traumatic effects of bullying have a profound impact on the lives of autistic people in the transition period, such as tertiary education and adulthood (104). Therefore, there is a need for a distinct policy outside the developmental disability frame, such as bullying of autistic and neurodivergent people who are excluded from registration. Moreover, Ministries of Education should develop education policies to enable autistic students into integrated education using universal designs and an interest-driven education system (105).

The findings also show the counterexample of the stereotypical portrayal of autistic traits. Autistic people are often portrayed as those who lack the theory of mind, do not have emotions, and cannot interact with society (106) because they are locked in their world. However, in the wake of the school violence, they were aware of the social attributes of the bullying, impressing their desire for counseling and improvement of the education system that was failing them.

The result negates previous research on teenage pupils’ attitudes toward people with disabilities (107), where classmates expressed more prosocial expression toward autistic pupils as time passed. Moreover, the result shows the negative effect when education authorities have few interventions for allistic persons to accept autistic people and people with disabilities. However, proper interventions for the pupils may make a difference (108). Therefore, education authorities should make methodologies to include autistic pupils in the integrated class, including the initiative of friendship with allistic pupils (109).

There have been some improvements because the researchers and participants were in school. From 2010, corporal punishment by teachers in schools was criminalized by “progressive superintendents” (97), and excessive use of corporal punishment has since been weakened in regulations. In 2021, the Civil Act of Korea was amended to prohibit corporal and emotional punishment at home. However, conservative political parties, media, and NGOs still view the ban on institutional violence in schools as a weakening of their authority and have called for its full reinstatement to increase “study ability” alongside night-time independent study (110). Parents still justify corporal and emotional punishment when they do not feel a sense of parenting efficacy (111). Korean society must recognize this fact: Student violence is reproduced by school violence to reinforce school authority (12).

## 6 Conclusion

This study reaffirms existing research that impresses bullying against autistic persons, views bullying as a social construction (112), and sheds light on the low quality of life among autistic people in Korea. 

In recent years, the airing of *The Glory* (2022) and the continuous revelation of allegations of bullying by children of candidates for high public office have led to a growing social consensus on punishing perpetrators of bullying in Korea. *The Extraordinary Lawyer Woo Young-woo* (2022) also mentioned bullying against autistic pupils through media, which Woo overcame. However, the bullying has been largely excluded from media coverages.

Korean secondary schools, where bullying against autistic persons in Korea mostly happens, promote a “profound” competition to advance to the tertiary education system. In this competition, autistic behavior is arguably redefined by some peers as a stigma rather than a diversity with the acquiescence of some school teachers. As a result, some autistic people remaining in mainstream classes in secondary schools are seen as abnormal; Even many autistic pupils who have attended mainstream classes in primary schools are pushed into special classes or special schools due to school maladjustment and bullying, resulting in the exclusion from education to take the CSAT. From the perspective of autistic people, excessive competition in the Korean secondary education system and school violence might cause irreparable damage to those autistic pupils, their low academic performance, in turn excluding them from post-secondary education and decent jobs (113).

Now we see an increasing number of autistic people entering higher education systems around the world (114), and some autistic experts and scholars are actively engaged in autism research and leading participatory research, as well as a surge in the number of autistic doctors and even psychiatrists (115). In this regard, the interdisciplinary findings of this study, based on the humanities and social sciences, reject the view that school violence against autistic people is fate and logical conclusion caused by their lack of social skills (116). The dehumanization of autistic people is a result of intentional or unintentional discrimination and stigma from society caused by the societal inability to accommodate them. The immediate demands of the study participants, which included the provision of a counseling system for victims of school violence and punishment of perpetrators, are the evidence of such accommodation, and the need for stronger countermeasures against school violence.

Nevertheless, many autistic-related parties stress that there are more important things to the “people with autism spectrum disorder” (117), like 24-h care for “people with severe developmental disabilities” (118); Such argument may block finding social stigma, barricades against autistic and neurodivergent people in the society, and eventually loses reciprocal benefit from an autistic-inclusive society. The result of this study reveals there are much more important problems for autistic people, suggesting further studies to figure out their current quality of life. We hope autism research shift its focus from the medical model of disability, which violates the Convention on the Rights of Persons with Disabilities, to the human rights model of disability (23).

The results also provide several insights for further research. First, the results of the study will justify further research needs on autistic bullying experiences, especially on the autistic person’s lived experience outside the European and North American Countries (119) and by Autistic researchers (120, 121). We hope that this article facilitates further representative and comparative studies on Asia and other countries.

Next, the study results suggest the connection between school bullying and emotional effects (122) and failure from the social lifecycle path, which is the need for wellness and a high quality of life for each autistic person. For example, Japanese researchers report a connection between autistic traits during *hikikomori* or isolated youth (123, 124), which could be a starting point for a study on the emotional effect of autistic persons with bullying experience.

Third, the results call attention to the relationship between bullying and the deprivation of self-determination rights of persons with an impairment in decision-making ability. “People with developmental disabilities”, which ‘the Act on Guarantee of Rights of, and Support for Persons With Developmental Disabilities’ defines as autistic persons and persons with intellectual disabilities, can be easily placed under guardianship in Korea, which could lead to substituted decision-making (125). School Bullying tends to deteriorate their decision-making ability, making it hard to implement supported decision-making (126). Therefore, we need more research on the effect of school bullying on the decision-making ability of people with developmental disabilities.

Finally, the result urges fast and meaningful policymaking to the (Korean) government, which includes intervention strategies or possible revision of acts to prevent school violence against Koreans with disabilities, especially autistic pupils, according to their expected barriers (127).

The results from this research have some limitations: First, most participants relate to the Korean autistic community, which includes many self-diagnosed autistic persons. Therefore, someone might deny the quality of this research, according to the disability registration system and developmental disabilities dogma prevalent in Korea. However, there was no quality difference between reports from registered and “liminal” autistic persons. The result of the study reaffirms the viewpoint of other researchers on autistic traits and autistic advocators (22, 75, 128, 129) to include “people with social communication” disabilities and other self-diagnosed autistic persons into the whole number of autistic people.

One participant reported bullying at the workplace, which may induce post-traumatic stress (130). They said that workplace bullying was more severe. However, because the research focused on bullying in Korean secondary education, we did not hear the bullying in the transition and adult periods. We hope to continue further studies to fill up the cognitional hole of the unknown: the whole life of Korean autistic people.

As this study was conducted with adults, the length of time between the bullying incident and the interview may raise questions about the reliability of memory recall. However, many participants complimented the objectivity of this research, and the interviewers’ comments provided constant reliability. Moreover, the difficulty of recruiting and interviewing adolescents requiring their parents’ consent under Korean law and research ethics were factors that led us to limit the study to adults. We hope there will be an additional expansive study, including Korean autistic teenagers and adults under the Korean disability sphere.

## Data availability statement

The original contributions presented in the study are included in the article/supplementary material. Further inquiries can be directed to the corresponding authors.

## Ethics statement

The studies involving humans were approved by Institutional Review Board of Hanyang University. The studies were conducted in accordance with the local legislation and institutional requirements. The participants provided their written informed consent to participate in this study.

## Author contributions

WY: Investigation, Writing – original draft, Writing – review & editing. JS: Investigation, Writing – original draft. CJ: Supervision, Writing – original draft, Writing – review & editing.
